# Comparative Genomics of the Bacterial Genus *Streptococcus* Illuminates Evolutionary Implications of Species Groups

**DOI:** 10.1371/journal.pone.0101229

**Published:** 2014-06-30

**Authors:** Xiao-Yang Gao, Xiao-Yang Zhi, Hong-Wei Li, Hans-Peter Klenk, Wen-Jun Li

**Affiliations:** 1 Key Laboratory of Biogeography and Bioresource in Arid Land, Xinjiang Institute of Ecology and Geography, Chinese Academy of Sciences, Urumqi, China; 2 Key Laboratory of Microbial Diversity in Southwest China, Ministry of Education and the Laboratory for Conservation and Utilization of Bio-Resources, Yunnan Institute of Microbiology, Yunnan University, Kunming, China; 3 The First Hospital of Qujing City, Qujing Affiliated Hospital of Kunming Medical University, Qujing, China; 4 Leibniz-Institute DSMZ-German Collection of Microorganisms and Cell Cultures, Braunschweig, Germany; 5 University of Chinese Academy of Sciences, Beijing, China; Wake Forest University School of Medicine, United States of America

## Abstract

Members of the genus *Streptococcus* within the phylum *Firmicutes* are among the most diverse and significant zoonotic pathogens. This genus has gone through considerable taxonomic revision due to increasing improvements of chemotaxonomic approaches, DNA hybridization and 16S rRNA gene sequencing. It is proposed to place the majority of streptococci into “species groups”. However, the evolutionary implications of species groups are not clear presently. We use comparative genomic approaches to yield a better understanding of the evolution of *Streptococcus* through genome dynamics, population structure, phylogenies and virulence factor distribution of species groups. Genome dynamics analyses indicate that the pan-genome size increases with the addition of newly sequenced strains, while the core genome size decreases with sequential addition at the genus level and species group level. Population structure analysis reveals two distinct lineages, one including Pyogenic, Bovis, Mutans and Salivarius groups, and the other including Mitis, Anginosus and Unknown groups. Phylogenetic dendrograms show that species within the same species group cluster together, and infer two main clades in accordance with population structure analysis. Distribution of streptococcal virulence factors has no obvious patterns among the species groups; however, the evolution of some common virulence factors is congruous with the evolution of species groups, according to phylogenetic inference. We suggest that the proposed streptococcal species groups are reasonable from the viewpoints of comparative genomics; evolution of the genus is congruent with the individual evolutionary trajectories of different species groups.

## Introduction

The genus *Streptococcus* comprises a wide variety of pathogenic and commensal gram-positive bacteria [1]. Pathogens and some commensals of *Streptococcus* show a surprising capacity for adaptation to new hosts and resistance to antibiotics and immune responses. As a result, they have caused the spread of infection and significantly increased morbidity and mortality rates all over the world, leading to huge health and economic loss [2]–[7]. A small group of commensals are opportunistic pathogens like *Streptococcus oralis*, while others are harmless saprophytes like *Streptococcus thermophilus* used as starter cultures in the food industry [8]. Due to the diversity and clinical importance of this genus, *Streptococcus* has attracted the attention of medical scientists and microbiologists and has undergone considerable taxonomic revision.

Previously, the taxonomy of the genus *Streptococcus* mainly focused on morphological, biochemical and serological characterization, but it is still not very clear with modern genomic data as yet not adequately considered [9]. Recent applications of chemotaxonomic approaches, genomic DNA-DNA hybridization and 16S rRNA sequencing techniques have not only provided significant insights into the natural relationships among streptococci, but have also influenced significantly their taxonomy and nomenclature [10]–[13]. These revisions form the basis of delineation and reveal the natural grouping of species into “species groups” [14]. The species groups have been named “Pyogenic”, “Mitis”, “Anginosus”, “Bovis”, “Mutans” and “Salivarius” respectively, and they encompass the majority of described species (several species remain ungrouped). Although these polyphasic taxonomy approaches are still widely used in many laboratories, limits of biochemical determination, and low efficiency operation of DNA hybridization [15] as well as possible phenotypic and ecological differentiation underlying identical 16S rRNA genes [16] all inevitably hamper the evolutionary and taxonomic investigations of streptococci. Moreover, understanding of the species groups relies on relevant biochemical features, so the reliability of species groups under a larger molecular data set needs to be determined. Hence, investigation of their phylogenetic relationships and evolutionary implications is necessary to enrich our knowledge of the evolution of the genus *Streptococcus*.

With increasing advances in sequencing and computational technologies, application of genomic tools has revolutionized microbial ecological studies and has drastically expanded our view on the previously underappreciated microbial world [17], [18]. In this context, the number of available streptococcal genomes is growing exponentially. Whole-genome sequencing has gained new insights into microevolution of streptococci, and also helped researchers to decipher their host adaptation [19], [20], determine virulence factors [21] and track pathogenesis mechanisms, laying the foundation for vaccine candidate development [22], [23]. Comparative genomics is primarily used to investigate intraspecies variation [24], [25], which is extended to the diversity studies of closely related *Streptococcus* species [26], [27]. As mentioned above, comparative genomic analyses of streptococci along with other bacteria have revealed microbial genomes as dynamic entities shaped by multiple forces, including genome reduction, genome rearrangements, gene duplication, and acquisition of new genes through lateral gene transfer [26], [28]. As a large number of bacterial genomes are sequenced, it has become increasingly evident that one strain’s genome sequence is not entirely representative of other members of the same species. Information from more genomes is needed to understand the dynamic nature of genomes, and to comprehend the evolutionary process at higher taxonomic levels [1], [29], [30]. Thus, evolution of the genus *Streptococcus* underscores the need to implement comprehensive whole-genome analyses with more extensive genomic sampling.

This study uses genomic data to explore the evolution of the genus *Streptococcus* within the context of proposed species groups. Here, we employ comparative genomic analyses of the genus *Streptococcus* to define the pan-genome and core genome, assess population structure, infer phylogenetic relationships and determine virulence factor distribution of species groups. Specifically, the analyses enabled us to test (1) pan-genome size and core genome size of *Streptococcus* and species groups; (2) the phylogenetic relationships among those groups based on genomic data; (3) the reasonableness of species groups raised by associated biochemical features and 16S rRNA gene analysis; and (4) distribution of virulence factor among species groups, in order to explore their implications in evolution of the genus.

## Materials and Methods

### 1 Materials

This study used 138 streptococcal genomes covering most species in the genus (Figure S1). Most of them were divided into 6 species groups according the previous studies [10], [11], [14]. Because *Streptococcus suis* has not been assigned to an existing species groups, we named it as the “Unknown” group. The genomic data was obtained from genome release in the public database NCBI (ftp://ftp.ncbi.nlm.nih.gov/genomes/Bacteria/) as of May, 2013, including all complete genomes as well as draft genomes of type strains or strains for which a complete genome was not available. Characteristics of *Streptococcus* species and strains were acquired from NCBI (http://www.ncbi.nlm.nih.gov/genome) and JGI (http://genome.jgi.doe.gov/) as well as related genome publications [9], [31]–[33].

### 2 Methods

#### 2.1 Identification and functional classification of homologous clusters

Homologous clusters used for subsequent analyses were determined by the program OrthoMCL version 2.0 [34]. In our analyses, all extracted protein sequences were adjusted to a prescribed format and were grouped into homologous clusters using OrthoMCL based on sequence similarity. The BLAST reciprocal best hit algorithm [35] was employed with 50% match cutoff and 1e-5 e-value cutoff, and Markov Cluster Algorithms (MCL) [36] were applied with an inflation index of 1.5. As a result, a matrix describing the genome gene content for 138 strains was constructed. The total 274,822 protein-coding genes were grouped into 18,528 homologous clusters, including common genes represented by 369 core homologous clusters. The functional category of each core homologous cluster was determined by performing BLAST program against Cluster of Orthologous Groups (COGs) database (http://www.ncbi.nih.gov/COG/) with 50% identity cutoff and 1e-5 e-value.

#### 2.2 Pan-genome, core genome and unique genes

In order to predict the possible dynamic changes of genome size at the genus and species group levels, the sizes of pan-genome, core genome and unique genes were simulated. 18,528 clusters, from OrthoMCL program, were parsed by Perl scripts. Then pan-genome (gene repertoire), core genome (common genes, mutually conserved) and unique genes (specific genes, only found in one genome) [37] were estimated as done in previous studies [38]–[41]. For pan-genome analysis starting from one single genome to 138 genomes, genomes were added 1000 times in a randomized order without replacement at each fixed number of genomes, and the gene repertoire was accumulated. The statistical analyses of core genome and unique genes followed the above procedures. Gene accumulation curves describing the dynamic changes of gene repertoire, common genes and new genes with the addition of new comparative genomes were implemented by SigmaPlot version 12.5. Furthermore, we employed best fitting functions to predict possible distributions of pan-genome, core genome as well as unique genes for streptococci, using the median values as determined by IBM SPSS Statistics version 19 [42].

#### 2.3 Population structure

In order to investigate the population structure of the genus *Streptococcus* and its relationships with species groups, the Markov chain Monte Carlo (MCMC) based program Structure version 2.3.4 [43], [44] was used to cluster individuals into populations. Initially, we treated orthologous genes as MLST sequence data from Extended FASTA Format into the Structure Format using xmfa2struct (available from http://www.xavierdidelot.xtreemhost.com/clonalframe.htm). The admixture ancestry model with assumption of correlated allele frequencies among populations was used. We ran the simulation 10 times under a burn-in period of 100,000 and a run length of 1,000,000 MCMC, without prior population information. K values from 1 to 7 were tested to allow us to identify the best K value, represented by the highest value of K and DK [45]. Results of the ten independent runs were averaged for each K value to determine the most likely model, i.e., the one with the highest likelihood, and they were subsequently plotted using Distruct version 1.1 [46]. The identification of the best K was evaluated following the DK-method through online program Structure Harvester (available at: http://taylor0.biology.ucla.edu/struct_harvest/) [47].

#### 2.4 Phylogenetic Analysis

To determine the phylogenetic relationships among *Streptococcus* species and species groups based on genomic data, both supermatrix and gene content methods were applied to infer phylogenetic trees. For the supermatrix method, we selected a set of orthologous genes shared by all 138 streptococcal strains (278 genes present in a single copy in all strains) according to the identification of homologous clusters. For each orthologous cluster, protein sequences were aligned using ClustalW version 2.1 [48] and the resulting alignments of individual proteins were concatenated to infer the organismal phylogeny using Neighbor-Joining (NJ) in MEGA version 5.20 [49] and the maximum likelihood algorithm (ML) in RAxML version 7.3.0 [50]. For the gene content method, a gene content matrix was parsed using a phyletic pattern indicating the presence (1) or absence (0) of the respective genes of all streptococcal strains. Jaccard distance (one minus the Jaccard coefficient) between pairwise genomes was calculated based on the gene content matrix. Hierarchical clustering (unweighted pair group method with arithmetic mean, UPGMA) in package PHYLIP version 3.6 [51] was employed to reconstruct the gene content dendrogram, using paired Jaccard distances.

#### 2.5 Virulence factor determination

To explore the distribution of species group-specific virulence factors, we collected all streptococcal virulence factors in the Virulence Factor Database (VFDB, http://www.mgc.ac.cn/VFs/). The relevant gene sequences of virulence factors were extracted from genomes, and all the protein-coding sequences of 138 *Streptococcus* strains analyzed were incorporated as the database. The virulence factor distribution for 138 streptococcal genomes was determined by BLAST with 50% match cutoff, 50% coverage cutoff and 1e-5 e-value cutoff. In cases of shared homologous genes related to virulence factors, phylogenetic trees were inferred by ML algorithms in RAxML version 7.3.0.

## Results and Discussion

### 1 Genomic size and GC content of species groups

According to previous studies on 16S rRNA gene sequence analysis and associated biochemical features of the genus *Streptococcus* (Table S1 in File S1) [10]–[12], 138 *Streptococcus* strains were divided into seven species groups: “Pyogenic”, “Mitis”, “Anginosus”, “Bovis”, “Mutans”, “Salivarius” and “Unknown” (Table S2 in File S1). The genome size varied from 1.64 Mb (*Streptococcus peroris* D1) to 2.43 Mb (*Streptococcus salivarius* D1) with the average value of 2.05 Mb. Within species groups, the genome size range and average showed no significant variations: Pyogenic (range 1.75–2.27 Mb, average 2.00 Mb), Mitis (range 1.64–2.39 Mb, average 2.07 Mb), Anginosus (range 1.82–2.29 Mb, average 1.96 Mb), Bovis (range 1.74–2.38 Mb, average 2.12 Mb), Mutans (range 1.92–2.42 Mb, average 2.09 Mb), Salivarius (range 1.8–2.43 Mb, average 2.01 Mb), and Unknown (range 1.98–2.23 Mb, average 2.10 Mb). Streptococcal genome size is relatively small when compared to other bacteria, and may indicate an adaptation for reproductive efficiency or competitiveness for a new host environment [52]. The genus *Streptococcus* is a low GC content taxon, and genomic GC content of its representatives range from 33.79% (*Streptococcus urinalis* D2) to 43.40% (*Streptococcus sanguinis* W1) with an average of 39.25%. Genomic GC content results from mutation and selection [53] involving multiple factors, including environment, symbiotic lifestyle, aerobiosis, nitrogen fixation ability, and the combination of polIIIa subunits [54].

### 2 Distribution and identification of homologous clusters

Homologous genes evolve through two fundamentally different ways, either through speciation events (producing orthologs) or by gene duplication events (producing paralogs) [55]. A clear distinction between orthologs and paralogs is critical for the construction of a robust evolutionary classification of genes and reliable functional annotation of newly sequenced genomes [56]. In this study, 274,822 protein coding sequences from 138 genomes of streptococci were grouped into 18,528 homologous clusters, including 8,203 clusters unique to one proteome. Of the 274,822 proteins, the majority had homologous counterparts; however, some proteins were unique and could not be matched to any homologs in the pan-genome of *Streptococcus* (Table S3 in File S1). The 18,528 homologous clusters included both orthologous clusters and paralogous clusters, and a histogram of the number of clusters vs. the number of genomes was bimodal, with maxima at those present in only one genome and those present in all 138 streptococcal genomes (Figure S2A). 274,822 proteins including orthologous proteins and paralogous proteins across 138 streptococcal genomes also provide the same result (Figure S2B). The broad orthologous/paralogous cluster here is composed of both absolute and relative parts, namely orthologs/paralogs and semi-orthologs/paralogs (accessory genes). The number of orthologs within each streptococcal genome is 278 and the percentage ranges from 11.24% (*Streptococcus ictaluri* D1) to 17.90% (*Streptococcus salivarius* D3). However, the number of paralogs within each streptococcal genome is not constant, ranging from 3.84% (95, *S. ictaluri* D1) to 6.25% (97, *S. salivarius* D3). Therefore, the percentage of core genes ranges from 15.08% (373, *S. ictaluri* D1) to 24.15% (375, *S. salivarius* D3) (Table S4 in File S1). The percentage of unique proteins shows obvious differences in each genome, therefore the view of stable genomes that function as unchanging information repositories has given way to a more dynamic view in which genomes frequently lose genes and incorporate foreign genes [57], [58]. Notably, the accessory genes account for significant portions in streptococcal genomes, since strains from same species or strains from different yet closely related species will share common genes.

A logical speculation often made in studying pathogen evolution implies that most host-specific adaption is associated with bacterial species-specific genes [26]. Previous studies revealed that there have been significant amounts of positive selection pressure on core genome components and that this selection pressure has occurred disproportionately in certain lineages [26]. According to COG classification analysis of core clusters, possible functions of 369 core clusters were identified and subdivided into 20 subcategories (Figure S3). There are 3 subcategories in information storage and processing, 7 subcategories in cellular processes and signaling, 8 subcategories in metabolism, and 2 subcategories were poorly characterized. Information storage and processing category makes up 38.2% of clusters, whereas cellular processes and signaling as well as metabolism categories make up 19.0% and 26.1% of clusters, respectively. Most of these genes are related to the colonization, persistence, and propensity to cause disease in these organisms [26]. Moreover, the poorly characterized part accounted for 16.8% may be involved in specific adaptations that help streptococci survive in novel environments.

### 3 Pan-genome and core genome analyses

#### 3.1 Pan-genome

**Figure 1:**
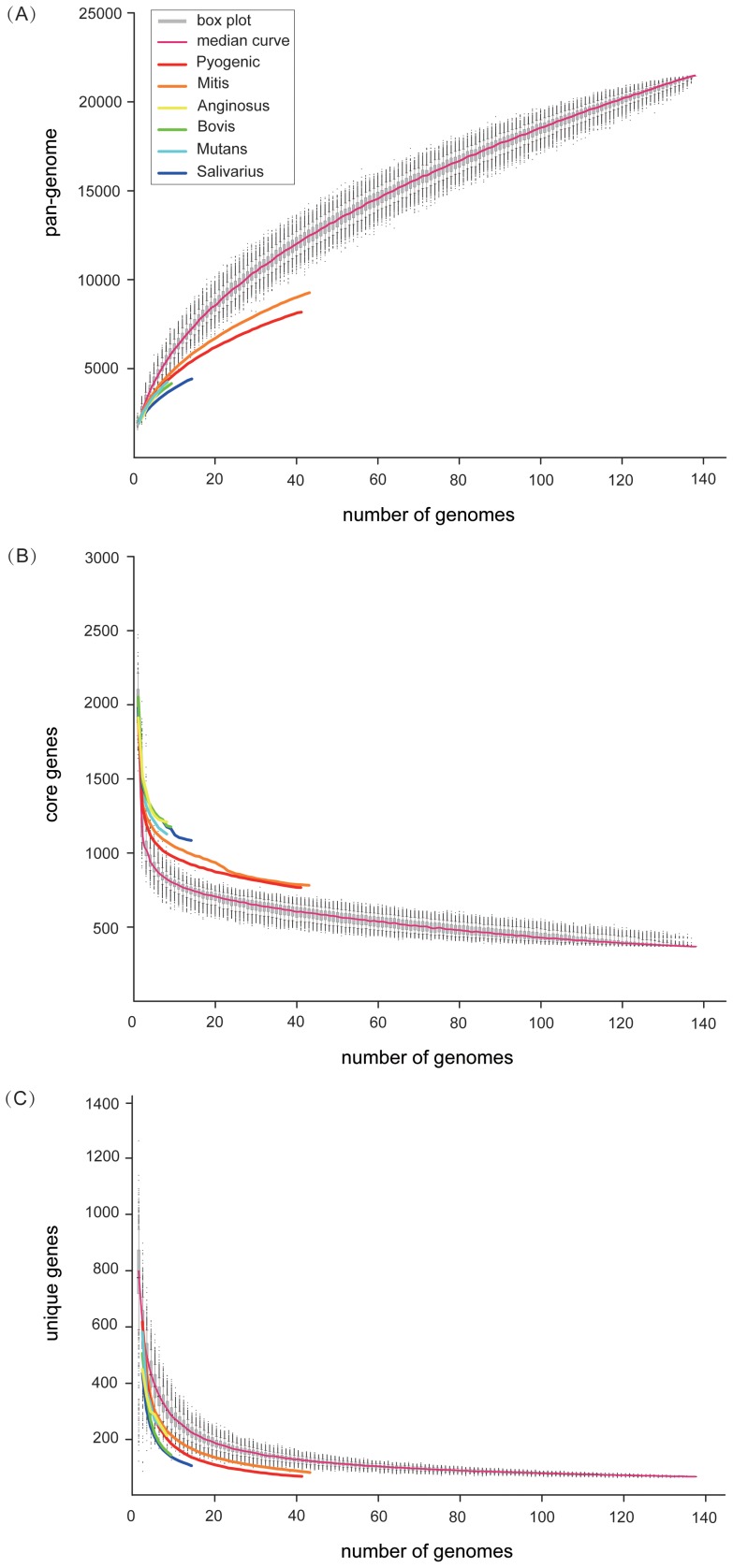
Size of pan-genome, core genome and unique genes for *Streptococcus.* (A) Total number of genes. The curve was fitted to the function 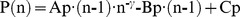 and parameters 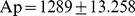, 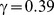, 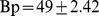, 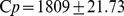
were determined under correlation 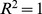. (B) Number of genes in common. The curve was fitted to the function 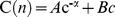. The best fit was obtained with correlation 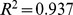 for 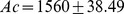, 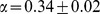, 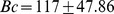 (C) Number of unique genes. The curve was fitted to the function 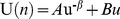, the best fit was obtained with correlation 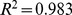 for 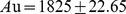, 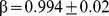, 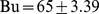. The upper and lower edges of the boxes respectively indicate 25 and 75 percentiles, and the horizontal carmine lines indicate 50 percentile under 1,000 different random input orders of genomes. The central vertical lines extend from each box as far as the data extends to a distance of at most 1.5 interquartile ranges. Colors represent Pyogenic (red), Mitis (orange), Anginosus (yellow), Bovis (green), Mutans (cyan), and Salivarius (blue) species groups, respectively.

Estimation of the *Streptococcus* pan-genome indicates that the gene repertoire steadily increased with sequential addition of each new genome, and tendency was opening until the last addition (Figure 1A). In this study, we predicted that the gene repertoire of the genus *Streptococcus* could hold at least 21,446 genes. There is a tremendous increase from the first addition to thirtieth addition and the growth gradually becomes gentle, with acquisition of only 51 genes after addition of the last genome. We performed a power law fitting with median values as described previously [38], [59] to model the possible trend and display the changing process through the function. The trend of streptococcal pan-genome size revealed that the genus possesses an open pan-genome for which the size increases with the addition of new sequenced strains. This was in accordance with previous studies on pan-genome of *Streptococcus* [24], [26], [27], [59], which indicated that the size of gene repertoire was underestimated and that the pan-genome size would continue to increase as more streptococcal genomes were sequenced.

In order to further verify that the pan-genome is open, the number of unique genes was calculated by incorporation of a new genome every time. In contrast to the pan-genome, the plot of new genes was fit well by a decaying function, and remarkably, the extrapolated curve reached an asymptotic value of 62, which meant that every newly sequenced genome could bring 62 new genes on average, even if many genomes were sequenced (Figure 1C). We therefore applied the exponential decay model to identify unique genes function using the median values. In light of the above analyses, we confirmed that the genus possesses an open pan-genome that increases in size with the addition of new sequenced strains. This was consistent with previous studies on the unique genes and pan-genome of *Streptococcus* [24], [26], [59], [60].

#### 3.2 Core genome

In contrast to pan-genome, estimation of the streptococcal core genome indicates that genes shared in all strains decreased with each addition, and it finally reached a plateau as the implication of keeping nearly constant over the last seven additions (Figure 1B). The decrease dropped from 1979 genes to 1179 genes at the first addition and kept stable at 369 genes since the next-to-last addition. As a result, the final constant of 369 shared genes was determined as the core genome size. The core gene number in each genome varied slightly because of involvement of duplicated genes and paralogs in shared clusters [59]. As observed for other bacterial species, the size of the *Streptococcus* core genome decreases as a function of genomes included, while the size of the pan-genome increases. The regression analysis of shared genes was extrapolated by fitting a decaying function, which was considered to provide the best fit to the dataset. Core and dispensable genes represent the essence and the diversity of the species, respectively [37]. As pointed out, this set of core genes does not correspond to the minimal set of genes necessary for an organism to survive and thrive in nature [5]. It is a backbone of essential components on which the rest of the genome is built [61].

The average gene content for *Streptococcus* genomes is 1,991±169 genes and thus the core genome accounts for less than a fifth of the average gene content, and only 9.3% of the estimated pan-genome. In addition, this clear variability of gene content between species was also evident in comparison across strains of the same species. It once again implies the obvious genomic plasticity among streptococci living in different habits and possessing diverse lifestyles [1], [62]. An open pan-genome is typical of those species that colonize multiple environments and have multiple ways of exchanging genetic material.

#### 3.3 Genomes of Species groups

Estimations for genome sizes of six species groups were simultaneously carried out, except the “Unknown” group (Figure 1). The trends of the core genome, pan-genome and unique genes in these species groups were similar to those trends at the genus-level as described above. However, sizes of pan-genome and new genes of species groups were obviously smaller than the one at genus-level after each addition, and core genome sizes of species groups were larger than the one at genus-level after each addition. Moreover, there were subtle differences in genome sizes among species groups after each addition. This may be due to the fact that various species with diverse genome sizes were subsumed into different species groups. For example, Pyogenic and Mitis include more species and have more unique genes, and thus occupy a larger proportion of pan-genome than other species groups.

### 4 Population structure

**Figure 2:**
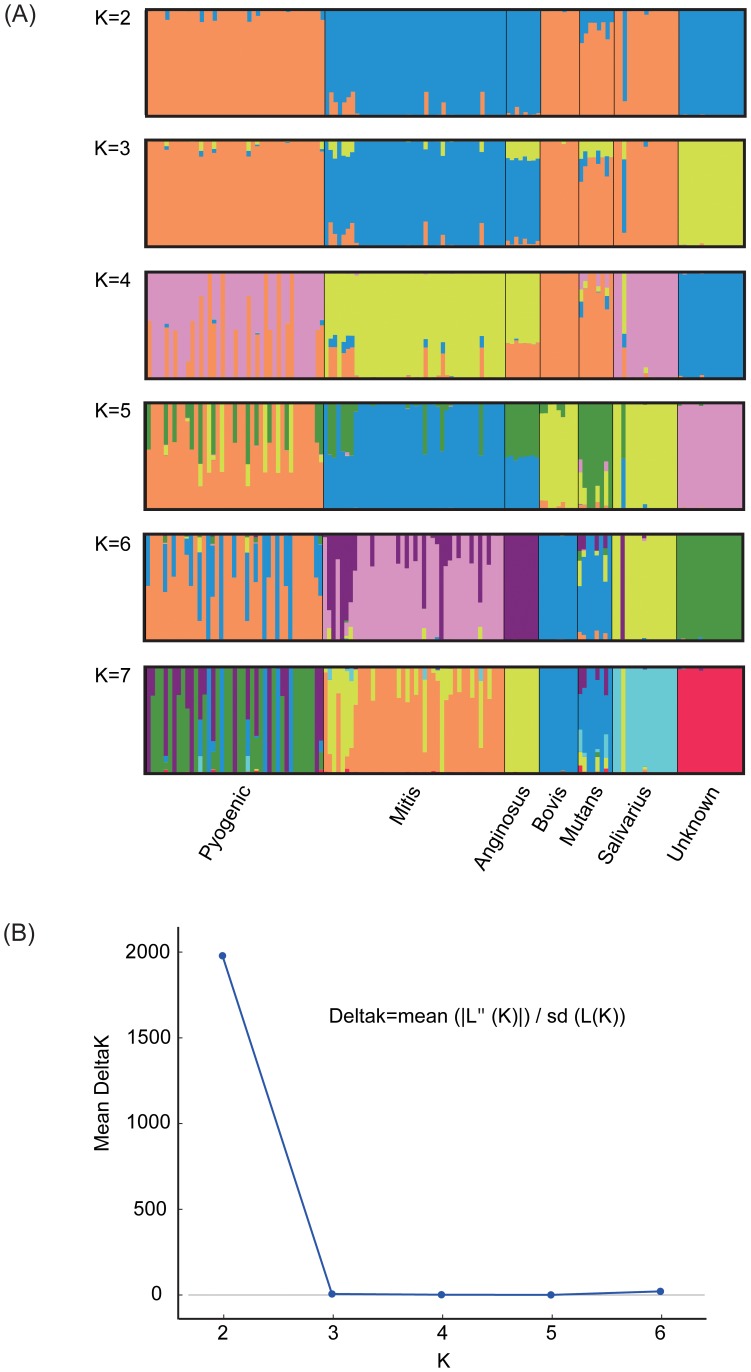
Population structure of streptococcal species groups. (A) The population memberships of the inspected species groups for a priori defined number of clusters K = 1–7 inferred by the Structure software. Each individual is represented by a thin vertical line divided into K colored segments that represent the individual’s estimated membership fractions in K clusters. Black lines separate individuals of different populations. Populations are labeled below the figure. (B) The detection of the true number of clusters inferred by the Structure software and set 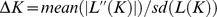 as a function of K. ΔK attains its highest value when K = 2, generated by Structure, according to Evanno et al.

The highest ΔK value (an ad hoc quantity related to the second order rate of change of the log probability of data with respect to the number of clusters) inferred from analysis using the program Structure [45] emerged when K = 2 (Figure 2B), indicating that streptococcal strains investigated here fall into two distinct populations (Figure 2A). The first of these populations included Pyogenic, Bovis, Mutans and Salivarius (orange color), and the second included Mitis, Anginosus and Unknown (blue color). Mutans appears to be a hybrid between the orange population and the blue population, with all individuals showing nearly 20% ancestry from the blue population composited of Mitis, Anginosus and Unknown. Mitis also shows fragmentary evidence for hybridization; genes from population including Pyogenic, Bovis, Mutans and Unknown were mixed into Mitis. The structures of two populations throw lights on evolutionary scenarios for streptococci and the relationships between populations and species groups.

### 5 Phylogenomic analyses

**Figure 3:**
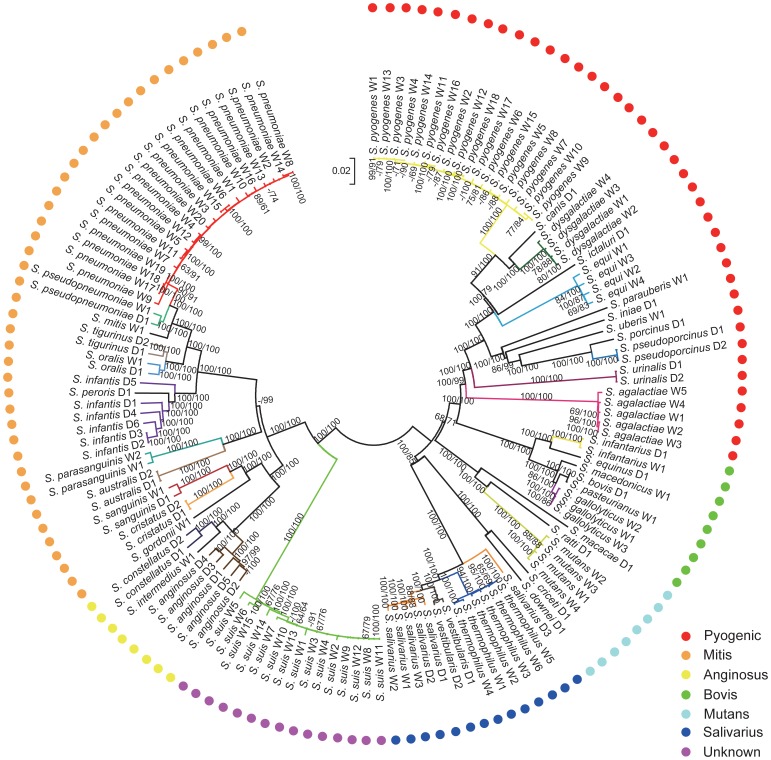
Phylogenomic tree of *Streptococcus*. The supermatrix tree was constructed based on maximum likelihood (ML, bootstrap value indicated as numerator) and neighbor-joining (NJ, bootstrap value indicated as denominator) algorithms, using a concatenated alignment of 278 orthologous proteins. All the 138 *Streptococcus* strains analyzed were assigned to the corresponding species groups and were marked with related colored circles. Different color-coded branches denoted different species.

The inferred phylogeny of *Streptococcus* based on analysis of 138 genomes had a well-supported, consistent topology under Neighbor-joining both (NJ) and Maximum Likelihood (ML) algorithms (Figure 3). Strains within the same species clustered together, regardless of whether the data was derived from complete or draft genomes. Similarly, genomes from the same species group clustered together. Clearer phylogenetic relationships can be acquired through more extensive genomic sampling, particularly analyzing the whole set of conserved genes across a taxonomical level such as the genus level. Additionally, core genomes will shed light on evolutionary and functional relationships among the related species [63]. The existence of a core set of genes present in all bacteria is a testament to the conservative nature of evolution. Within several billion years of bacterial evolution, no successful replacement of the core genes evolved in any of the lineages leading to the studied genomes. The core set of genes is under high positive pressure for functions that prevent drastic changes.

**Figure 4:**
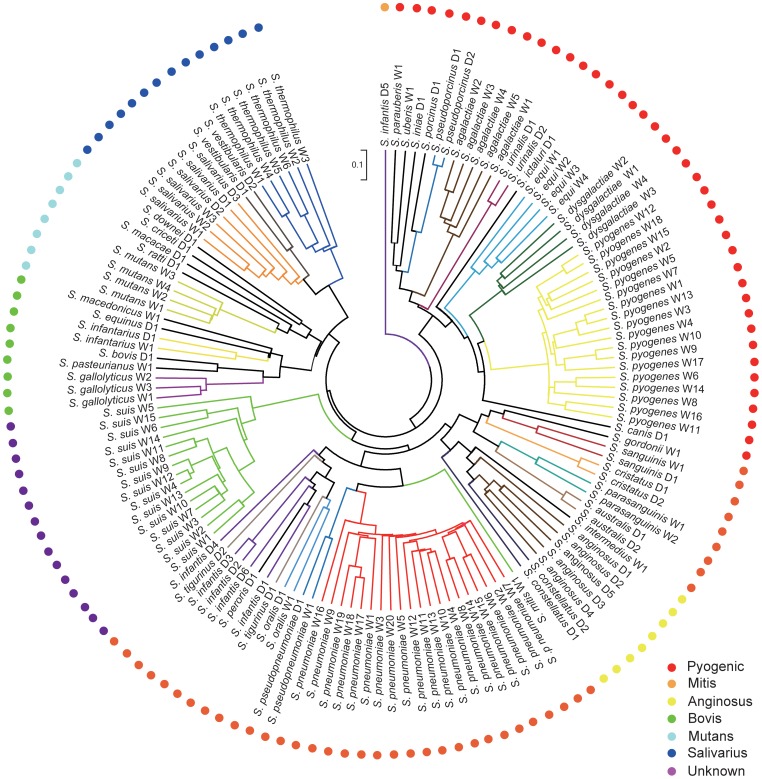
Gene content dendrogram of *Streptococcus*. The dendrogram was constructed by hierarchical clustering (UPGMA) based on the dissimilarities in gene content among 138 *Streptococcus* strains, using paired Jaccard distances which range from 0 to 1. Different color-coded branches denoted different species.

**Figure 5:**
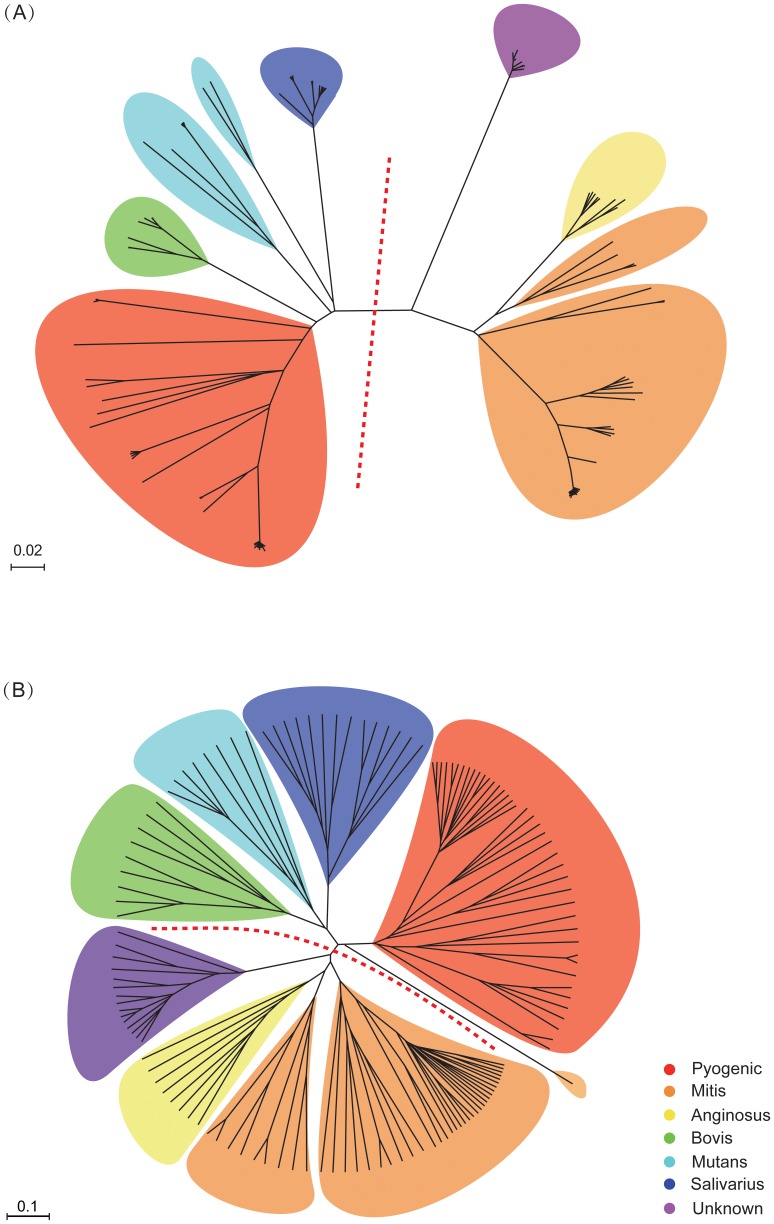
Phylogenomic relationships of streptococcal species groups. The clustering results of seven species groups were based on phylogenomic tree and gene content dendrogram. Each species group was painted with the assigned color as the above analysis.

The relationships among species and species groups were better understood from a gene content dendrogram (Figure 4), which used unweighted pair group method with arithmetic mean (UPGMA) algorithm [64]. Similar to the supermatrix tree, nearly all strains from the same species and most species from the same group cluster together. *Streptococcus infantis* D5 did not cluster with the other five strains in this species, which was likely caused by variation in gene composition as a result of gene annotation bias. The two dendrograms identify two main clades of species groups in accordance with the above Structure analysis (Figure 5A–B), one of which includes species from Mitis, Anginosus and Unknown, while the other one includes species from Salivarius, Mutant, Bovis and Pyogenic. To verify these relationships, we inferred the gene content dendrogram based on the core genome using UPGMA algorithm. The dendrogram topology based on the pan-genome most resembles that based on the core genome. Particularly, *Streptococcus infantis* D5 was incorporated into the Mitis species group, due to the fact that species-specific genes were removed and only shared genes were used for analysis (Figure S4A–B). The streptococci from Mutans are associated with dental plaque in human and animals. Here, Mutans group was divided into two subgroups, because this group overall is regarded as relatively loose with the member species having deep lines of descent [9]. Lateral gene transfer and recombination of genes have played a significant role in generating diversity in both Mitis and Anginosus species groups [65]–[68]. Species from Mitis and Anginosus have a close relationship with one another, consistent with the suggestion that Mitis and Anginosus formed subgroups within a single “Oralis group” according to the classification of Schleifer and Kilpper-Balz [12]. Therefore, hybridization between populations of clusters identified in Structure analysis can effectively explain the polyphyly in the phylogenetic tree.

### 6 Distribution of virulence factors

Virulence factors of pathogenic bacteria, such as streptococci, play an important role in conquering various niches through infecting hosts and adapting to new environments. Particularly fascinating is the fact that some bacterial species can invade tissues and elicit different diseases by expression of different combinations of virulence factors. Therefore, we further compared the *Streptococcus* genomes with respect to virulence gene content to uncover additional insights into the biology and evolution of this genus. The determination of virulence factors in *Streptococcus* was investigated on the basis of VFDB, and virulence factors were mainly distributed in 135 representatives of the streptococci (Table S5 in File S1). Particularly, all of the streptococci have a number of genes associated with capsule production, which plays a significant role in immune evasion. Abundant production of capsular polysaccharide composed of hyaluronic acid results in mucoid strains of group A *Streptococcus* associated with outbreaks of acute rheumatic fever [69]. *S. pneumonia* strains with capsule quickly colonize and multiply because of their ability to evade phagocytosis, whereas *strains* lacking capsule suffer phagocyte killing [70].

**Figure 6:**
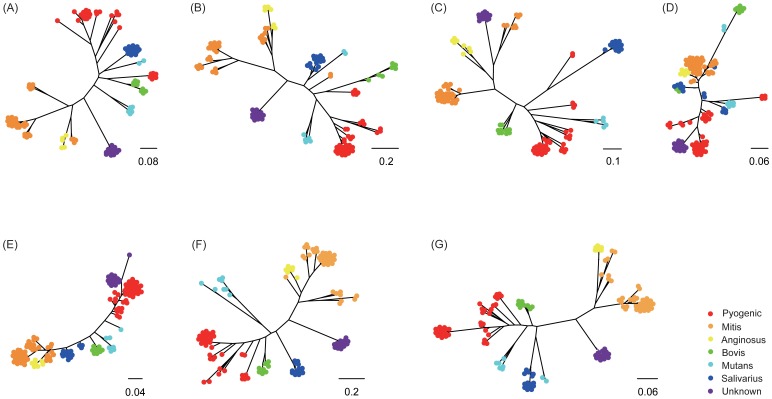
Phylogenetic dendrograms of seven conserved genes related to virulence factors. A, B, C and D represent trees of *pavA*, *srtA*, *slrA*, *plr/gapA* from adhesion factor, respectively; E represents tree of *eno* from exoenzyme factor; F and G represent trees of *htrA/degP* and *tig/ropA* from protease factor, respectively.

The prevalent pathogens like *Streptococcus agalactiae*, *S. mutans*, *S. pneumonia*, *S. pyogenes* and *S. suis* possessed abundant genes related to virulence factors, and an obvious regular distribution of virulence factors among species groups was not discovered. Seven relatively prevalent virulence genes were selected to construct ML phylogenetic trees to reveal the evolution of virulence (Figure 6). The seven virulence genes used were *pavA* (fibronectin binding proteins), *srtA* (sortase A), *slrA* (streptococcal lipoprotein rotamase) and *plr/gapA* (streptococcal plasmin receptor/GAPDH) from adhesion, *eno* (streptococcal enolase) from exoenzyme, *htrA/degP* (Serine protease) and *tig/ropA* (trigger factor) from protease, respectively. Interestingly, the phylogenetic relationships from five genes (Figure 6A–C, F–G) share a similar topology in accordance with the phylogenomic analyses. This implies that evolution of adhesion genes (i.e., *pavA*, *srtA* and *slrA*) and protease genes (i.e., *htrA/degP* and *tig/ropA*) is in concordance with evolution of the genus, and these virulence genes are generally monophyletic within most species groups. In contrast, Anginosus and Mitis are not fully resolved, and sometimes are monophyletic with the Unknown group. Thus, the evolutionary relationships of virulence between Unknown group and other groups are needed to investigate in future studies.

The tree topologies of *eno* and *plr/gapA* (Figure 6D–E) are different from the topologies of the other five virulence genes, which indicate phylogenetic clusterings incongruent with the proposed species clusters. Enolase of prokaryotic pathogens represents a multifunctional protein involved in glycolytic and plasminogen binding and activation [71]. Also, it plays a crucial role in fibrinolysis, homeostasis and the degradation of extracellular matrix (ECM) [72]–[74], enabling infection of tissues and migration between organs. Enolases from *Ureaplasma* and *Mycoplasma* were found to be more similar to archaebacterial enolases than to their bacterial counterparts [75]. Besides, lateral transfer events between endosymbiont and apicomplexan account for evolution of cryptomonad and chlorarachniophyte algal enolases [76]. Genetic exchange of enolases between streptococci and hosts could account for phylogram of enolase being incongruent with those of other markers. Streptococcal plasmin receptor, namely, glyceraldehyde-3-phosphate dehydrogenase (GAPDH) constitutes a protein family which displays diverse activities in different subcellular locations, in addition to its well-characterized role in glycolysis [77]. GAPDH of streptococci has been reported to bind fibronectin, lysozyme, the cytoskeletal proteins myosin and actin, affecting colonization of those bacteria [78]. LGT events have been frequently documented in the evolution of GAPDH [79], [80]. Interestingly, both enolase and GAPDH are two main receptors of plasminogen in streptococci, and more efforts are required to enlighten their origin.

## Conclusions

Applications of chemotaxonomic approaches, DNA hybridization and 16S rRNA gene sequencing have resulted in the proposal of “species groups” for streptococci with various lifestyles. Our study, using population structure, phylogenetic and phylogenomic analyses of 138 *Streptococcus* genomes, offers additional insights into the evolution of species and species groups within this genus. Population structure of streptococcal species groups indicated that all *Streptococcus* strains branched into two distinct populations, with Pyogenic, Bovis, Mutans and Salivarius species groups forming one population, and Mitis, Anginosus and Unknown groups clustering into another population, suggesting that there are two major evolutionary lineages within this genus. Phylogenetic relationships based on core genome and pan-genome suggest that species from the same group are close to each other and indicate a pattern of different species groups accompanying the evolution of the genus *Streptococcus*, which is in accordance with the population structure analysis and provides supports for the proposed species groups based on comparative genomics approaches. Identification of virulence factors in streptococci revealed the toxin essence of highly pathogenic streptococci. Moreover, several virulence factors evolve in the same way as species groups according to phylogenies of their common virulence genes. All analyses indicate that the evolution of streptococci is congruent with the evolutionary pattern of species groups. The genus *Streptococcus* possesses an open pan-genome, thus the size of the pan-genome is yet underestimated and will increase as additional streptococcal strains are sequenced. Although the estimated genome size meshes with previous studies cited in the analysis, limitations in our abilities to accurately estimate genome size variation also limit the robustness of our inferences. These inferences should be accepted with caution and, as hypotheses, remain open for testing and refinement in future studies using dataset with more comprehensive sampling of streptococcal strains from a broader habitat range. Nonetheless, this study provides insights into streptococcal species differentiation and enriches our knowledge of evolution within the genus *Streptococcus*.

## Supporting Information

Figure S1 Phylogenetic tree of the genus *Streptococcus* based on 16S rRNA gene sequences. The phylogenetic tree was constructed based on ML (bootstrap values on the left of slashes) and NJ (bootstrap values on the right of slashes) algorithms. Species with red fonts had genome data and were analyzed in this study. Species with asterisks possessed complete genome sequences.
(TIF)

Figure S2 Occurrence of homologous clusters and proteins within 138 *Streptococcus* proteomes ranged from 1 to 138. (A) At one extreme of the horizontal axis are the species-specific clusters (8344, 45.03%), while at the opposite end of the scale are clusters, which include genes from every proteome (369, 1.99%). (B) At one extreme of the horizontal axis are the species-specific proteins present in a single proteome (8582, 3.12%), while at the opposite end of the scale are situated the genes found in all 138 proteomes (51318, 18.67%).
(TIF)

Figure S3 Histogram of core gene clusters assigned COG functional categories. COG categories are indicated to the right of the figure. The ordinate axis indicates the individual COG sub-categories for orthologous and paralogous clusters. The horizontal axis indicates the number of clusters assigned to each COG sub-category.
(TIF)

Figure S4 Comparison of phylogenetic relationships of seven species groups. The clustering results of seven species groups were obtained from gene content dendrograms using different dataset: (A) pan-genome and (B) core-genome.
(TIF)

File S1 **Table S1,** Classification of streptococcal species groups based on biochemical characteristics. **Table S2,** Genomic size and GC content of *Streptococcus* species and species groups. **Table S3,** Complete list of the 18,528 homologous clusters in 138 *Streptococcus* genomes. **Table S4,** Homologous genes proportion and distribution of *Streptococcus* species and species groups. **Table S5,** Determination of virulence factors in *Streptococcus*.
(XLS)
