# Supplementary figures and images for: Comparative Genomics of the Bacterial Genus Streptococcus Illuminates Evolutionary Implications of Species Groups
Source: PLoS One. 2014 Jun 30;9(6):e101229. doi: 10.1371/journal.pone.0101229 (PMC4076318; doi:10.1371/journal.pone.0101229)

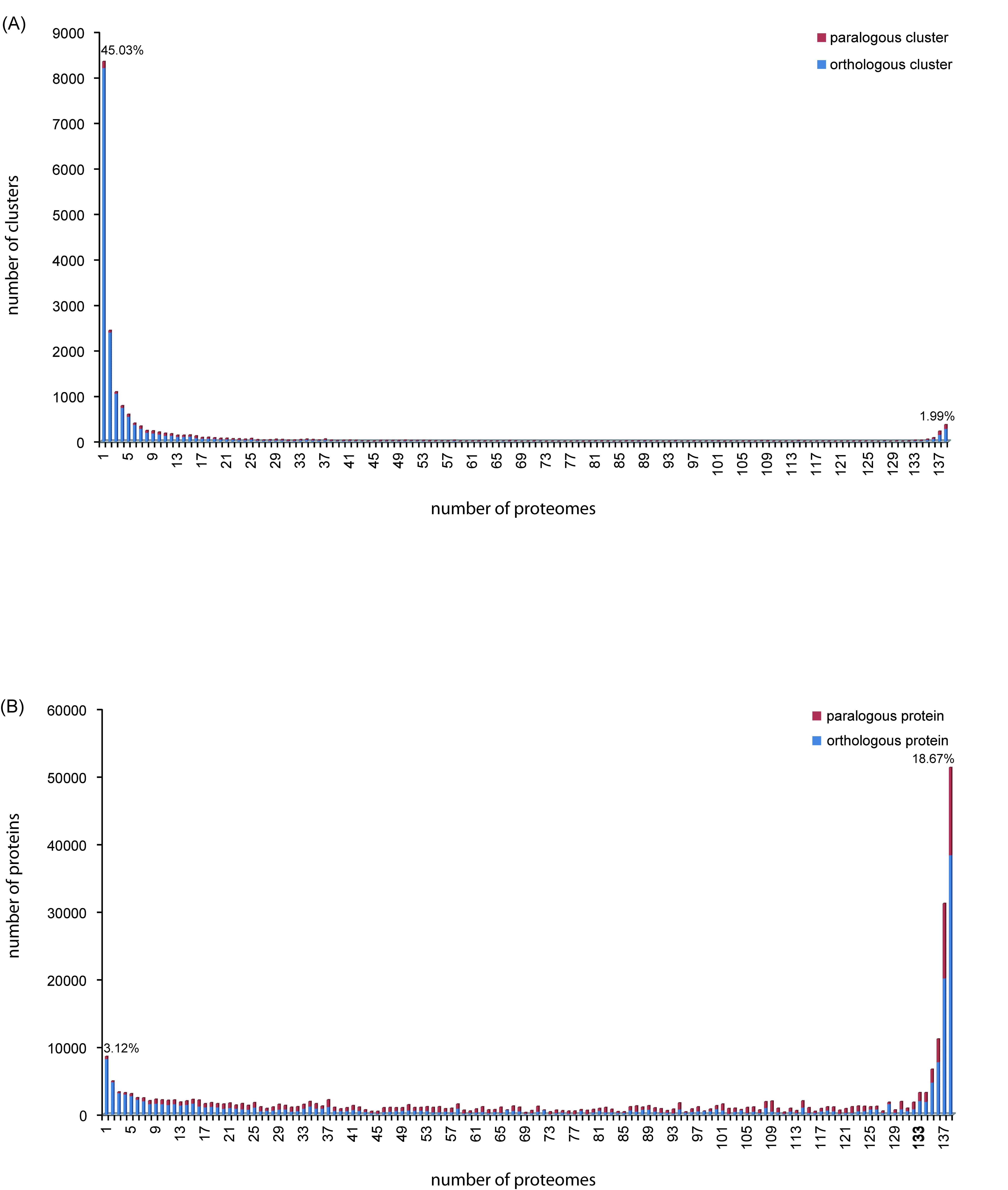

Supplement: Figure S2 — Occurrence of homologous clusters and proteins within 138 Streptococcus proteomes ranged from 1 to 138. (A) At one extreme of the horizontal axis are the species-specific clusters (8344, 45.03%), while at the opposite end of the scale are clusters, which include genes from every proteome (369, 1.99%). (B) At one extreme of the horizontal axis are the species-specific proteins present in a single proteome (8582, 3.12%), while at the opposite end of the scale are situated the genes found in all 138 proteomes (51318, 18.67%). (TIF) [file pone.0101229.s002.tif]

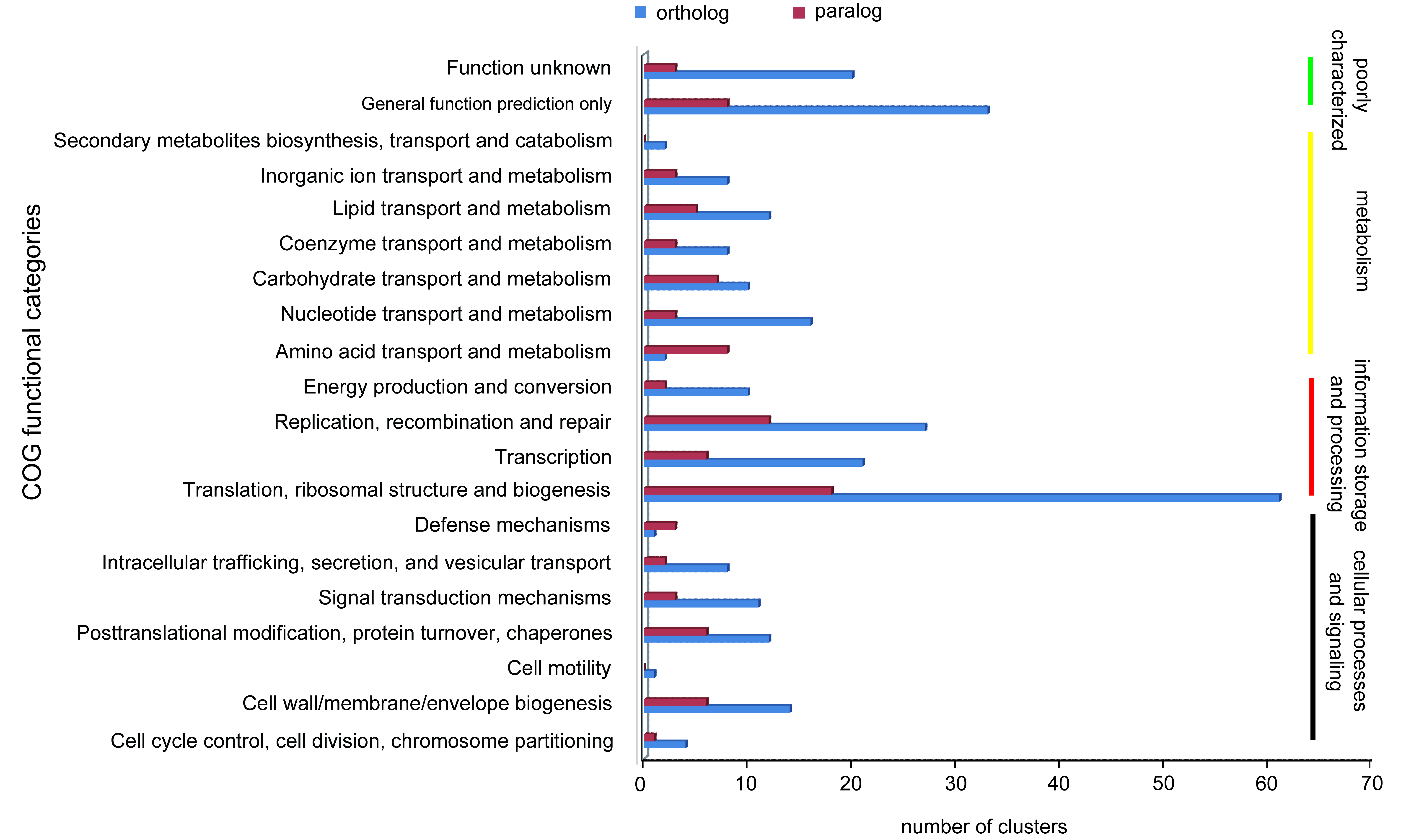

Supplement: Figure S3 — Histogram of core gene clusters assigned COG functional categories. COG categories are indicated to the right of the figure. The ordinate axis indicates the individual COG sub-categories for orthologous and paralogous clusters. The horizontal axis indicates the number of clusters assigned to each COG sub-category. (TIF) [file pone.0101229.s003.tif]

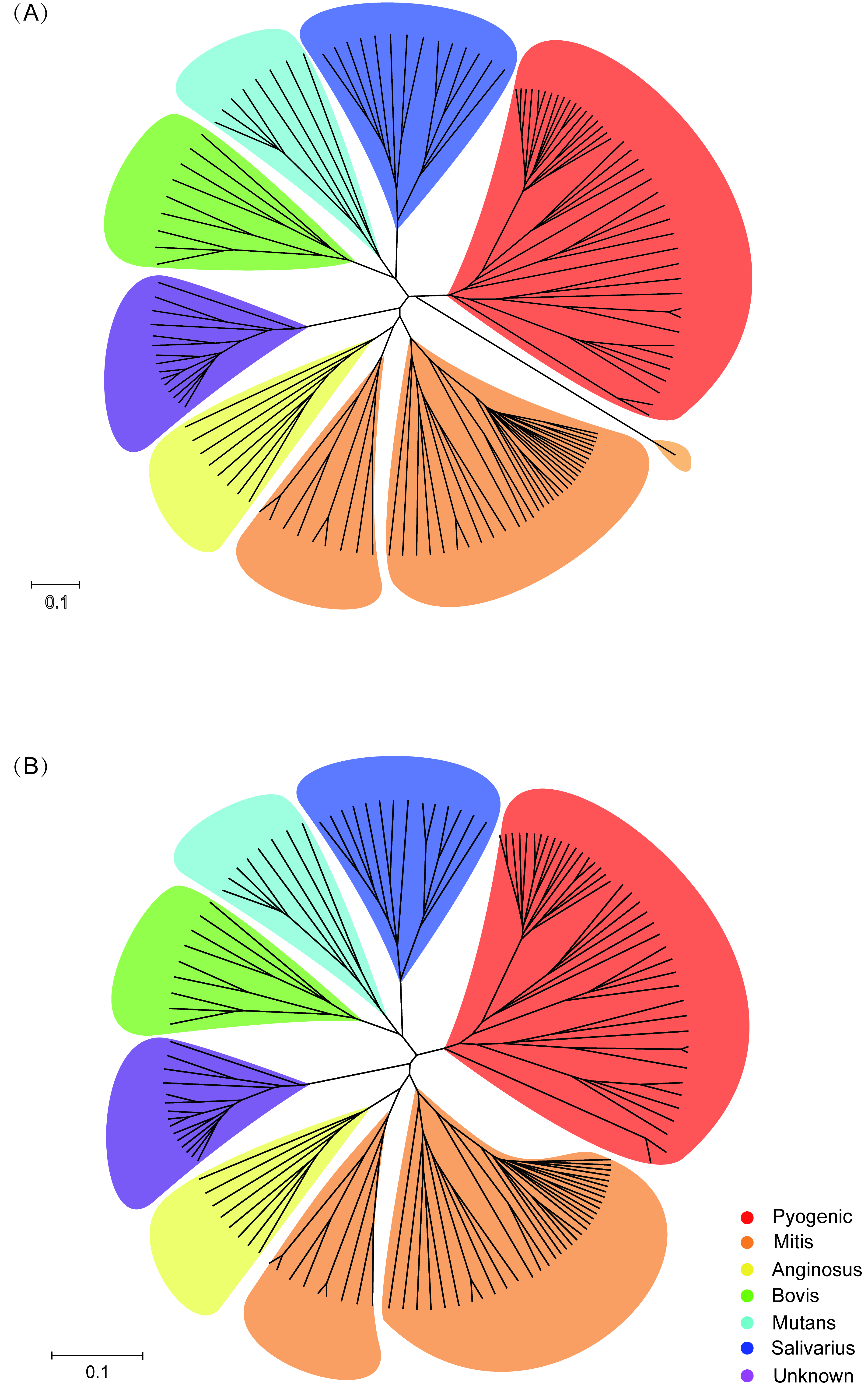

Supplement: Figure S4 — Comparison of phylogenetic relationships of seven species groups. The clustering results of seven species groups were obtained from gene content dendrograms using different dataset: (A) pan-genome and (B) core-genome. (TIF) [file pone.0101229.s004.tif]
